# Enhancement of Blood–Brain Barrier Permeability and Delivery of Antisense Oligonucleotides or Plasmid DNA to the Brain by the Combination of Bubble Liposomes and High-Intensity Focused Ultrasound

**DOI:** 10.3390/pharmaceutics7030344

**Published:** 2015-09-21

**Authors:** Yoichi Negishi, Masaya Yamane, Naho Kurihara, Yoko Endo-Takahashi, Sanae Sashida, Norio Takagi, Ryo Suzuki, Kazuo Maruyama

**Affiliations:** 1 Department of Drug Delivery and Molecular Biopharmaceutics, School of Pharmacy, Tokyo University of Pharmacy and Life Sciences, 1432-1 Horinouchi, Hachioji, Tokyo 192-0392, Japan; E-Mails: y091215@toyaku.ac.jp (M.Y.); y104085@toyaku.ac.jp (N.K.); endo@toyaku.ac.jp (Y.E.-T.); y114099@toyaku.ac.jp (S.S.); 2 Department of Applied Biochemistry, School of Pharmacy, Tokyo University of Pharmacy and Life Sciences, 1432-1 Horinouchi, Hachioji, Tokyo 192-0392, Japan; E-Mail: takagino@toyaku.ac.jp; 3 Laboratory of Drug and Gene Delivery Research, Faculty of Pharma-Sciences, Teikyo University, 2-11-1 Kaga, Itabashi-ku, Tokyo 173-8605, Japan; E-Mails: r-suzuki@pharm.teikyo-u.ac.jp (R.S.); maruyama@pharm.teikyo-u.ac.jp (K.M.)

**Keywords:** blood–brain barrier, bubble liposomes, ultrasound, nucleic acid delivery

## Abstract

The blood–brain barrier (BBB) is a major obstacle that prevents therapeutic drugs or genes from being delivered to the central nervous system. Therefore, it is important to develop methods to enhance the permeability of the BBB. We have developed echo-contrast gas (C_3_F_8_) entrapping liposomes (Bubble liposomes, BLs) that can work as a gene delivery tool in combination with ultrasound (US) exposure. Here, we studied whether the permeability of the BBB can be enhanced by the combination of BLs and high-intensity focused ultrasound (HIFU). Mice were intravenously injected with Evans blue (EB). BLs were subsequently injected, and the right hemispheres were exposed to HIFU. As a result, the accumulation of EB in the HIFU-exposed brain hemispheres was increased over that observed in the non-HIFU-exposed hemispheres, depending on the intensity and the duration of the HIFU. Similarly, the combination of BLs and HIFU allowed fluorescent-labeled antisense oligonucleotides to be delivered into the HIFU-exposed left hemispheres of the treated mice. Furthermore, a firefly luciferase-expressing plasmid DNA was delivered to the brain by the combination method of BLs and HIFU, which resulted in the increased gene expression in the brain at the focused-US exposure site. These results suggest that the method of combining BLs and HIFU together serves as a useful means for accelerating the permeability of BBB and thereby enabling antisense oligonucleotides or genes to be delivered to the focused brain site.

## 1. Introduction

In today’s rapidly aging society, there is an urgent need for the development of breakthrough treatments for refractory central nervous system (CNS) diseases such as Alzheimer’s disease or Parkinson’s disease. However, there is still no strategy for curing these diseases. It is expected that gene therapy or nucleic acid therapy (e.g., siRNA, miRNA, or antisense oligonucleotides) will continue to be key methods for treating CNS diseases. However, the delivery of therapeutic genes or nucleic acids into the brain parenchyma is limited because of the presence of the blood–brain barrier (BBB), which prevents therapeutic molecules from crossing into the brain [1]. Recently, the gene expression in CNS tissues by the local gene delivery with viral-vectors or non-viral vectors via intracranial injection has been demonstrated, but their gene expressions are shown in limited brain regions [2,3,4,5]. These direct injection methods are also highly invasive, and the risk increases when the treatments are repeated. On the other hand, with systemic injection methods, nanoparticles as non-viral vectors including liposomes, micelles, and dendrimers can be delivered to their brain tissues by utilizing the significant change of the BBB integrity, however, there is room for the improvement of their diffusion to reach target tissues. Therefore, development of nanocarriers for treating CNS diseases has been focused on achieving efficient systemic delivery of therapeutic genes by using targeting molecules and functional materials [6,7].

Among non-viral physical delivery methods [8,9,10], it has been shown that ultrasound (US) exposure improves drug or gene delivery efficiency into tissues and cells, a technique known as sonoporation [9]. US is believed to disrupt cell membrane and cause transient pores in the cell membrane by US cavitation activity, enabling the entry of extracellular drug or gene into viable cells [8,11,12,13]. Focused US (FUS)-mediated microbubbles, which are known as an echo-contrast gas agent for medical US imaging, are used to enhance gene transfection efficiency after US-induced cavitation while reducing cellular damage [14,15,16,17]. It has been also reported that using FUS-mediated microbubbles enables a transient BBB opening in the localized brain region [18]. In addition, submicron-sized bubbles (nanobubbles), which are smaller than conventional microbubbles and are expected to reach deeper tissue, were recently reported [19].

Polyethylene glycol (PEG)-modified liposomes, useful nanocarriers of drugs, antigens, and genes, can be easily prepared in a variety of sizes and modified to add a targeting function [20,21,22,23]. Therefore, we have previously reported PEG-modified liposomes encapsulating echo-contrast (C_3_F_8_), “Bubble liposomes” (BLs), which are submicron-sized (approximately 500 nm) and have the potential of a novel gene delivery carrier in combination with ultrasound (US) exposure [24,25,26,27,28,29].

In the present study, we examined whether the permeability of the BBB can be enhanced by the combination of BLs and high-intensity focused ultrasound (HIFU), thereby enabling the delivery of nucleic acids (e.g., antisense oligonucleotide) or plasmid DNA expressing the luciferase gene into the brain at a focused US exposure site.

## 2. Experimental Section

### 2.1. Preparation of Bubble Liposomes (BLs)

The Bubble liposomes were prepared using previously described methods [24,26]. Briefly, PEG liposomes composed of 1,2-dipalmitoyl-*sn*-glycero-3-phosphocholine (DPPC) (NOF Corporation, Tokyo, Japan) and 1,2-distearoyl-*sn*-glycero-3-phosphatidyl-ethanolamine-polyethylene glycol (DSPE-PEG_2000_-OMe) (NOF Corporation, Tokyo, Japan) in a molar ratio of 94:6 were prepared via a reverse-phase evaporation method. In brief, all reagents were dissolved in 1:1 (*v*/*v*) chloroform/diisopropyl ether. Phosphate-buffered saline was added to the lipid solution, and the mixture was sonicated and followed by evaporation at 47 °C. The organic solvent was completely removed, and the size of the liposomes was adjusted to less than 200 nm using extruding equipment and a sizing filter (pore size: 200 nm) (Nuclepore Track-Etch Membrane, Whatman plc, Kent, UK). The lipid concentration was measured using a Wako phospholipid C test (Wako Pure Chemical Industries, Ltd., Osaka, Japan). The BLs were prepared from liposomes and perfluoropropane gas (Takachio Chemical Ind. Co. Ltd., Tokyo, Japan). First, 2 mL sterilized vials containing 0.8 mL liposome suspension (lipid concentration: 1 mg/mL) were filled with perfluoropropane gas, capped, and then pressurized with an additional 3 mL perfluoropropane gas. The vial was placed in a bath-type sonicator (42 kHz, 100 W) (BRANSONIC 2510j-DTH, Branson Ultrasonics Co., Danbury, CT, USA) for 5 min to form the BLs.

### 2.2. Ultrasound Experimental Setup

Sonitron HIFU 5000 (NEPAGENE, Inc., Chiba, Japan) was used as a transducer. The HIFU was generated by a 3.5 MHz single element transducer (NEPAGENE, Inc., Chiba, Japan) with a diameter of 30 mm and a radius of 50 mm. The focal zone had a diameter and length of 1.1 and 16 mm. Ultrasound transmission gel was used to cover the area between the transducer and the mouse’s skull to maximize the transmission of the ultrasound. The right hemisphere brain was exposed to HIFU.

### 2.3. Extravasation of Evans Blue Dye (EB)

Extravasation of Evans blue dye (EB) were performed as described methods in previous report [30,31]. Briefly, male ICR mice (5 weeks old) were intravenously injected with Evans blue dye (EB) (100 mg/kg), which stably binds with albumin in the blood. After 5 min, BLs (100 μg/mouse) were also injected, and the right hemispheres were exposed with a 3.5 MHz pulsed HIFU (10% duty, 10–60 s) with different intensities (0.5–1.5 kW/cm^2^). After several hours, the treated mice were infused intravenously with PBS as a perfusion medium using a syringe pump at a constant speed. The mice were perfused with PBS via the left ventricle. After the perfusion and brain removal, the brains were then divided into the right and left hemispheres before measuring the amount of EB that was extravasated. The non-exposed left hemispheres of the treated mice were used as the control. The samples were weighed, soaked in formamide solution, and incubated for 24 h at 55 °C. Subsequently, the extracted dye concentration was determined using a spectrophotometer at 620 nm. To understand the effect of BLs with HIFU exposure on the duration of BBB permeability at the focused site, the mice were intravenously injected with BLs, and the right hemispheres were exposed to HIFU. After 5 min, 30 min, 3 h or 24 h, the mice were intravenously injected with EB. The amount of EB extravasation in each brain was examined after 3 h after the EB injection, as described above.

### 2.4. Delivery of Fluorescent-Labeled Dextrans

The mice were injected in the tail vein with Bubble liposomes (100 mg/mouse) and exposed to HIFU with 3.5 MHz, 10% Duty, 1.5 kw/cm^2^, 60 s. After 5 min, each mouse was injected with fluorescent-labeled dextrans (70 or 2000 kDa: 100 mg/mouse). Fluorescent-labeled dextrans were purchased from Sigma–Aldrich (St. Louis, MO, USA). After three hours, the treated mice were infused intravenously with PBS and then 4% PFA as a perfusion medium using a syringe pump at a constant speed. The mice were perfused with PBS and 4% PFA via the left ventricle. After the perfusion and brain removal, each brain was fixed in 4% PFA at 4 °C overnight and immersed in 30% sucrose/0.1 M PBS at 4 °C overnight. The fixed brain was embedded in an optimal cutting temperature compound (Sakura Finetek, Co. Ltd., Tokyo, Japan) and frozen at −80 °C. Each brain section was cut into slices 200 μm thick that were mounted on a poly-l-lysine coated slide. The sections were evaluated by fluorescence microscopy (KEYENCE: BZ8100). The nuclei were stained with DAPI. For quantification of the fluorescent-labeled dextran, the treated brains were then divided into the right and left hemispheres. The cell lysates and tissue homogenates were prepared in lysis buffer (0.1 M Tris–HCl (pH 7.8), 0.1% Triton X-100, and 2 mM EDTA). Subsequently, the extracted fluorescence intensity was measured at excitation wavelengths of 493 nm with emission at 520 nm.

### 2.5. Delivery of Phosphorodiamidate Morpholino Oligomers (PMOs)

The 3′-Carboxyfluorescein-labeled phosphorodiamidate morpholino oligomers (PMOs) M23D(+7–18: 5′-GGCCAAACCTCGGCTTACCTGAAAT-3′) were purchased from Gene Tools (Philomath, OR, USA). To determine the accumulation of PMOs in the whole brain, fluorescently labeled PMO (100 μg) and BLs (100 μg/mouse) were intravenously injected into male ICR mice (5 weeks old), and the right hemispheres were exposed to 3.5 MHz pulsed HIFU conditions (intensity: 1.5 kW/cm^2^, exposure time: 30 s). Three hours after the injection, the mice were euthanized. Each tissue was corrected, and the fluorescence intensity was observed by the Maestro *in vivo* imaging system (Kurabo, Industries, Ltd., Osaka Japan). Subsequently, the brain tissue was sectioned using a brain slicer (divided with 2 mm slices), and their tissues were examined using an *in vivo* fluorescence imaging system. The fluorescence was measured at excitation wavelengths of 445–490 nm with emission at 515 nm. To examine the accumulation of fluorescently labeled PMO in the brain, the treated mice were infused intravenously with PBS as a perfusion medium using a syringe pump at a constant speed. The mice were perfused with PBS via the left ventricle. After the perfusion and brain removal, each brain was fixed in 4% PFA at 4 °C overnight and immersed in 30% sucrose/0.1 M PBS at 4 °C overnight. The fixed brain was embedded in optimal cutting temperature compound (Sakura Finetek, Co. Ltd., Tokyo, Japan) and frozen at −80 °C. Each brain section was prepared with a thickness of 6 μm and mounted on a poly-l-lysine coated slide. The sections were evaluated by fluorescence microscopy (KEYENCE: BZ8100). The nuclei were stained with DAPI.

### 2.6. Delivery of Plasmid DNA into the Brain

A solution (200 μL) of BLs (100 μg/mouse) and plasmid DNA expressing the firefly luciferase gene (pcDNA3-Luc: 50 μg) was intravenously injected into male ICR mice (5 weeks old), and the right hemispheres were exposed to 3.5 MHz pulsed HIFU conditions (intensity: 1.5 kW/cm^2^, exposure time: 30 s). Twenty-four hours after the treatment, the mice were euthanized. The brains were then divided into the right and left hemispheres. The cell lysates and tissue homogenates were prepared in lysis buffer (0.1 M Tris–HCl (pH 7.8), 0.1% Triton X-100, and 2 mM EDTA). The luciferase activity was measured using a luciferase assay system (Promega, Madison, WI, USA) and a luminometer (LB96V, Belthold Japan Co. Ltd., Tokyo, Japan). The activity is indicated by relative light units (RLU) per mg of protein. For the *in vivo* imaging experiment on luciferase gene expression, the mice were anaesthetized and *i.p.* injected with d-luciferin (150 mg/kg) (Promega). After 10 min, luciferase expression was observed with an *in vivo* luciferase imaging system (IVIS) (Xenogen Corporation, Alameda, CA, USA). This *in vivo* imaging experiment was performed at 1–3 days after the delivery of plasmid DNA. To capture the *ex vivo* luciferase imaging, twenty-four hours after the delivery of plasmid DNA, the mice were euthanized. The tissues were collected, and the luciferase imaging was observed by the *in vivo* luciferase imaging system (IVIS) (PerkinElmer, Waltham, MA, USA). Subsequently, the brain tissue was sectioned using a brain slicer (divided into 2-mm slices), and their tissues were soak in d-luciferin solution (15 mg/mL) and examined using the *in vivo* luciferase imaging system (IVIS).

### 2.7. Evaluation of Tissue Damage

Male ICR mice (5 weeks old) were intravenously injected with BLs (100 μg/mouse), and the right hemispheres were exposed to 3.5 MHz pulsed HIFU conditions (intensity: 1.5 kW/cm^2^, exposure time: 60 s). The next day, the left ventricles in the treated mice were perfused with PBS and 4% paraformaldehyde (PFA) as a perfusion medium, using a syringe pump at a constant speed. Each brain was fixed in 4% PFA at 4 °C overnight and immersed in 30% sucrose/0.1 M PBS at 4 °C for 48 h. The fixed brain was embedded in an optimal cutting temperature compound (Sakura Finetek, Co. Ltd., Tokyo, Japan) and frozen at −80 °C. Each brain section was cut into slices 10 μm thick that were mounted on a poly-l-lysine coated slide. The section was processed by both hematoxylin and eosin (H&E). H&E staining was also performed on the brain sections using the standard technique. Each coronal section was viewed using a light microscope (KEYENCE: BZ8100).

### 2.8. In Vivo Studies

The animal use protocol and relevant experimental procedures were approved by the Committee of Animal Use and Welfare of Tokyo University of Pharmacy and Life Sciences on 6 June 2014 and 28 April 2015 (authorization number: 14-29 and 15-40). All experimental protocols for animal studies were conducted in accordance with the Principle of Laboratory Animal Care in Tokyo University of Pharmacy and Life Sciences.

### 2.9. Statistical Analysis

All data are shown as the mean ± SD (*n* = 3 to 6). The data were considered to be statistically significant when *p* < 0.05. A *t*-test was used to calculate the statistical significance.

## 3. Results and Discussion

### 3.1. Permeability of BBB by the Combination of BL and HIFU Exposure

**Figure 1:**
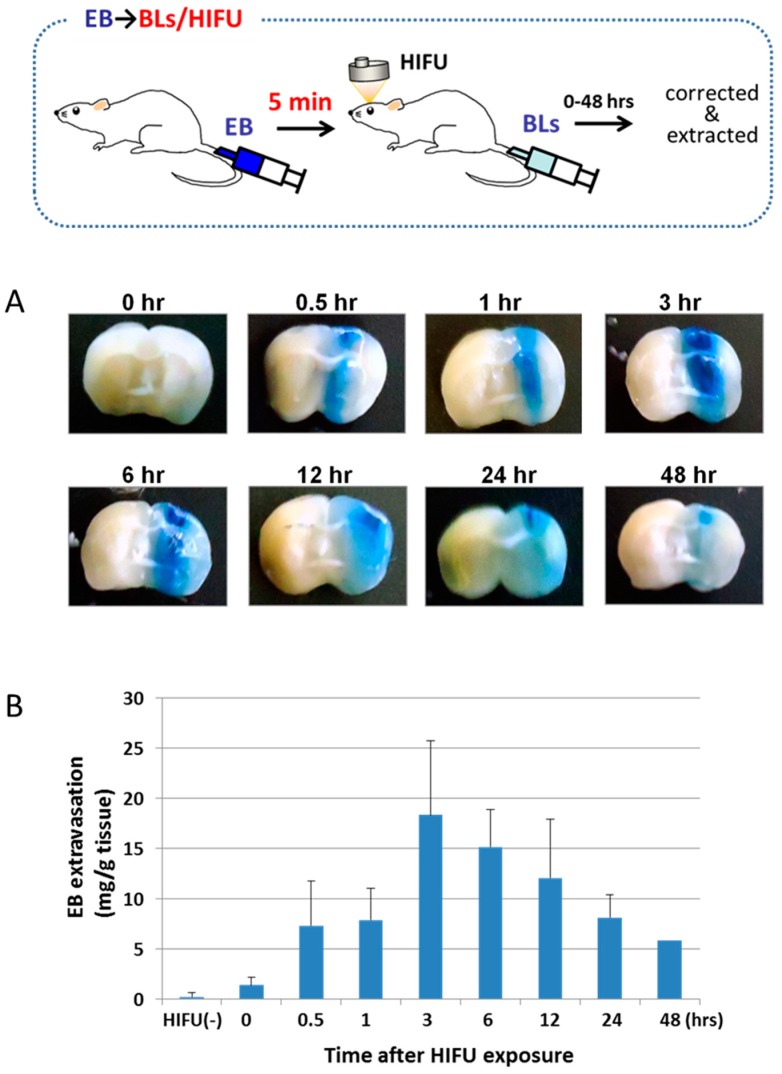
EB extravasation in the brain as a function of time after HIFU exposure. (**A**) Distribution of BBB disruption by extravasation of EB. Right brain: HIFU-exposed site. Left brain: control (non-exposed site). (**B**) The amount of EB extravasation in the right brain. The mice were injected into the tail vein with EB 100 mg/kg. After 5 min, they were injected with Bubble liposomes (100 μg/mouse) and exposed to HIFU with 3.5 MHz, 10% duty, 1.5 kw/cm^2^, and 60 s. After the treatment, each tissue was corrected at the indicated time. Each value represents the mean S.D. (*n* = 6). EB: Evans blue, BL: Bubble liposomes.

To study the permeability of the BBB by the combination of BLs and HIFU exposure, an EB uptake analysis was performed. It is well known that EB stably binds to serum albumin and that the observation of EB extravasation indicates albumin extravasation. Therefore, EB is also used to assess the permeability of blood vessels or the BBB [30,31]. Mice were intravenously injected with EB, BLs were subsequently injected, and the right hemispheres were exposed to HIFU. After several hours, the treated mice were perfused intravenously with PBS. The brains were then divided into the right and left hemispheres before observing the appearance and measuring the amount of EB extravasated. As shown in Figure 1a, the distribution of the BBB disruption by EB extravasation was locally observed at the HIFU-exposed site but not in the non-exposed site. The quantitative analysis showed that the amount of EB extravasation was highest at 3 h after the HIFU exposure and that the amount gradually decreased thereafter (Figure 1b). These results suggest that the BBB permeability can be enhanced through the treatment of BLs with HIFU.

**Figure 2:**
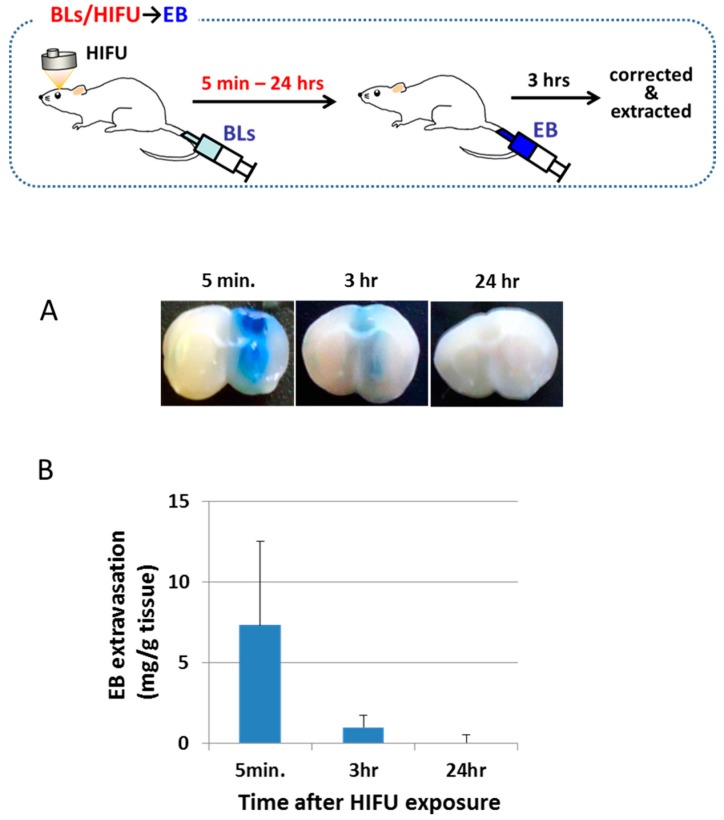
EB extravasation in the right brain (HIFU-exposed site) as a function of time after HIFU exposure. (**A**) Distribution of the BBB indicated by the extravasation of EB. Right brain: HIFU-exposed site. Left brain: control (non-exposed site). (**B**) The amount of EB extravasation in the right brain. The mice were injected into the tail vein with Bubble liposomes (100 μg/mouse) and exposed to HIFU with 3.5 MHz, 10% duty, 1.5 kw/cm^2^, and 60 s. After a definite period of time (5 min to 24 h), each mouse was injected with EB (100 mg/kg). After three hours, the right brain was corrected. Each value represents the mean S.D. (*n* = 4). EB: Evans blue, BL: Bubble liposomes.

The duration of the BBB permeability at the focused site after the treatment was then determined (Figure 2). Mice were intravenously injected with BLs, and the right hemispheres were exposed to HIFU. After 5 min, 30 min, or 24 h, mice were intravenously injected with EB. The amount of EB extravasation in each brain was examined after 3 h after the EB injection. This result shows that the permeability of the BBB in the focused site was significantly higher during the first 30 min compared with the other timespans. The EB extravasation declined to a lower level by approximately 3 h after the treatment of the BLs with HIFU exposure. In contrast, after 24 h, the EB extravasation was poor. These results suggest that the permeability of the BBB is reversed by 3 h after the treatment of BLs and HIFU exposure.

**Figure 3:**
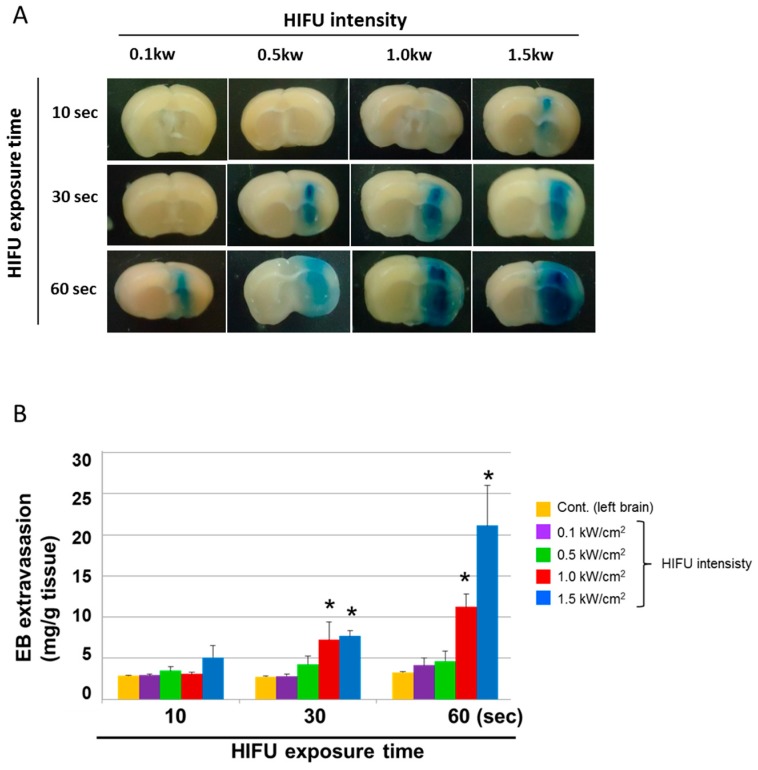
The amount of EB extravasation in the right brain (HIFU-exposed site). (**A**) Distribution of BBB disruption by extravasation of EB. Right brain: HIFU-exposed site. Left brain: control (non-exposed site). (**B**) The amount of EB extravasation in the right brain. The mice were injected into the tail vein with EB 100 mg/kg. After 5 min, the mouse was injected with Bubble liposomes (100 μg/mouse) and exposed to HIFU with 3.5 MHz, 10% duty, and 1.5 kw/cm^2^. After three hours, each tissue was corrected. The HIFU exposure times were 10, 30, or 60 s. The intensities were 0.1, 0.5, 1.0, or 1.5 kW/cm^2^. Each value represents the mean S.D. (*n* = 4). * *p* < 0.05 *vs.* cont. EB: Evans blue.

**Figure 4:**
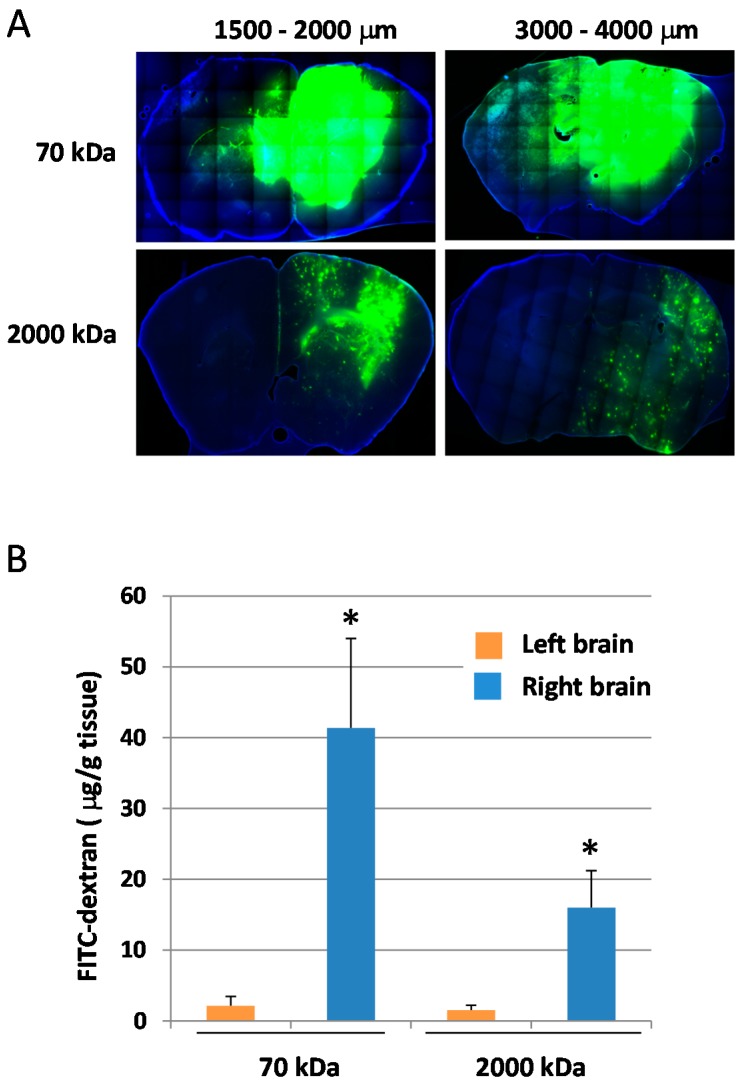
Delivery of fluorescent-labeled dextran into the brain by the combination of BLs and HIFU exposure. (**A**) Distribution of fluorescent-labeled dextran in coronal sections. Right brain: HIFU-exposed site. Left brain: control (nonexposed site). The mice were injected in the tail vein with Bubble liposomes (100 mg/mouse) and exposed to HIFU with 3.5 MHz, 10% Duty, 1.5 kw/cm^2^, 60 s. After 5 min, each mouse was injected with fluorescent-labeled dextrans (70 or 2000 kDa: 100 mg/mouse). After three hours, the brains were collected. The brain sections were sliced at 200 µm. The nuclei were stained with DAPI. Each specimen was observed by fluorescent microscopy. Each sectioned depth region from the frontal lobe of the cerebrum is shown above the corresponding photograph. (**B**) Quantification of fluorescent-labeled dextran. The treated brains were collected, and the fluorescent-labeled dextrans were extracted. Each value represents the mean S.D. (*n* = 4). * *p* < 0.05 *vs.* left brain.

**Figure 5:**
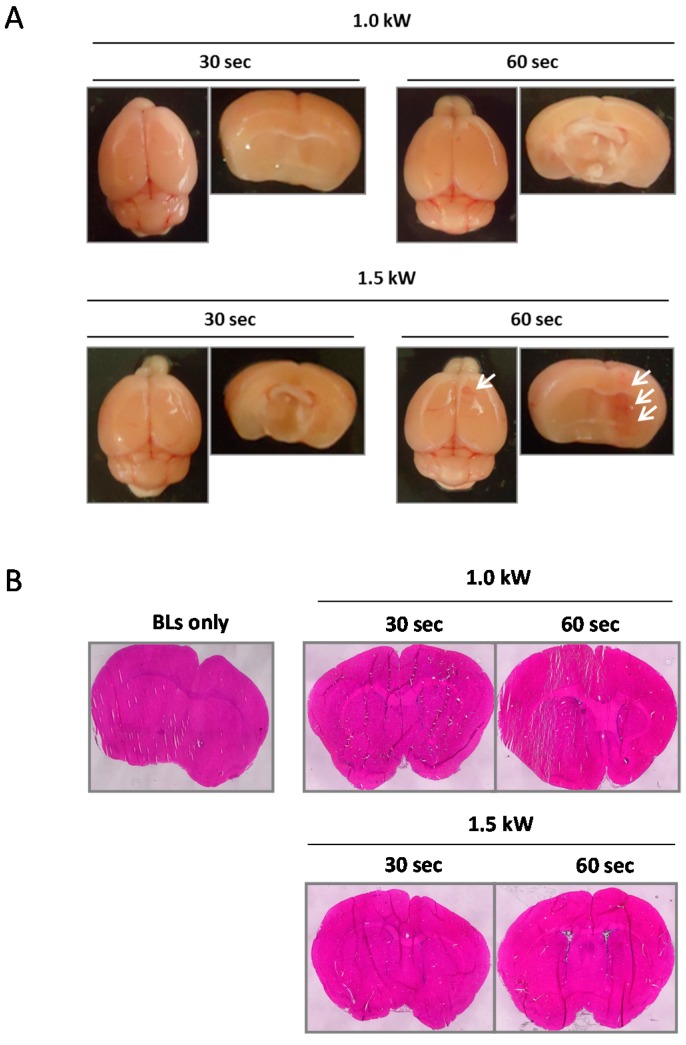
Histological analysis of brain after treatment with HIFU exposure. BLs were intravenously injected into mice, and then the right hemispheres were exposed to 3.5 MHz pulsed HIFU conditions (intensity: 1.0 or 1.5 kW/cm^2^, exposure time: 30 or 60 s). Twenty-four hours after the treatment, each brain was dissected. (**A**) The appearance of the whole brain and its coronal section. The white arrow indicates the region of erythrocyte extravasations in the exposure site. (**B**) H&E staining. Each brain section was sliced at 10 µm (*n* = 3). Each coronal section underwent H&E staining. Each specimen was observed using a light microscope. BL: Bubble liposomes.

We next examined the effect of BLs with HIFU exposure (especially with different exposure times and intensities) on the BBB permeability at the focused site. Mice were intravenously injected with EB; after 5 min, BLs were injected; then, the right hemispheres were exposed to different HIFU conditions (intensity: 0.1–1.5 kW/cm^2^, exposure time: 10–60 s). After three hours, each tissue was corrected. As shown in Figure 3A, the tissues’ appearances showed that the accumulation of EB at the focused site increased in HIFU conditions with higher intensities and longer exposure times. In addition, the faint accumulation of EB could be observed in the lower HIFU conditions (intensity: 1.5 kW/cm^2^; exposure time: 10 s). The quantitative analysis also shows that the permeability of the BBB in the focused site was dependent on the exposure time and HIFU intensity (Figure 3B). These results suggest that the BBB permeability can be enhanced after treatment with BLs and HIFU, or with the use of microbubbles, as reported previously [30,31]. We next examined the delivery of the fluorescent-labeled dextrans (70 and 2000 kDa) into the brain by the combination of BLs and HIFU exposure. As shown in Figure 4A, both of the dextrans with different sizes were distributed diffusely in the region of the focused right brain. However, the distribution level and amount of the 70 kDa dextran in the focused right brain were significantly higher than that of the 2000 kDa dextran (Figure 4B). In addition, the punctate fluorescence in the diffusion of the 2000 kDa dextran was in the focused right brain (Figure 4A). These results suggest that there is a size limitation for the molecule delivered using the combination method of BLs and HIFU and that the delivery efficiency of the molecule using this method is dependent on the size. We also performed histological analysis of brain tissue after treatment with BLs and HIFU exposure. As shown in Figure 5B, significant tissue damage could not be seen in the coronal section using H&E staining. However, in the appearance of the whole brain and its coronal sections, slight erythrocyte extravasations were rarely observed in the right hemispheres exposed to HIFU conditions (intensity: 1.5 kW/cm^2^, exposure time: 60 s) (Figure 5A). Therefore, relatively lower power (short exposure time: 30 s) was applied in the subsequent delivery experiments.

### 3.2. Delivery of Phosphorodiamidate Morpholino Oligomers (PMOs) into the Brain Using a Combination of BLs and HIFU Exposure

**Figure 6:**
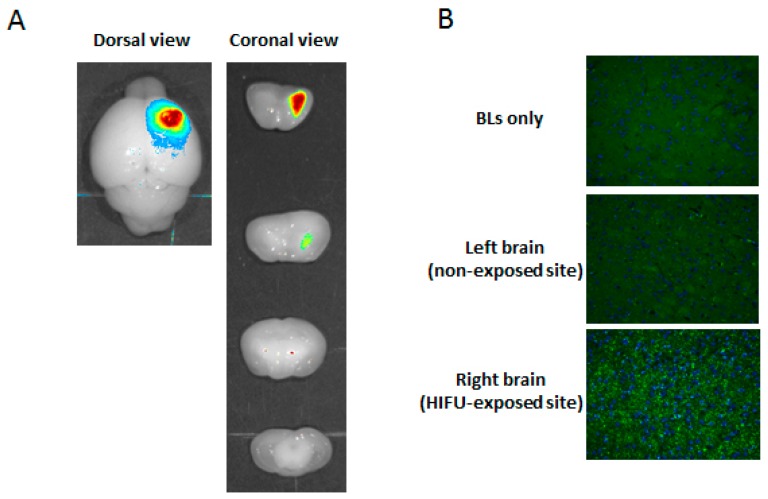
Delivery of fluorescent-labeled PMOs into the brain by the combination of BLs and HIFU exposure. (**A**) *Ex vivo* brain imaging of fluorescent-labeled PMO. Fluorescent-labeled PMO and BLs were intravenously injected into the mice, and then the right hemispheres were exposed to 3.5 MHz pulsed HIFU conditions (intensity: 1.5 kW/cm^2^, exposure time: 30 s). Three hours after the treatment, the brains were collected and observed using the Maestro *in vivo* imaging system. (**B**) Fluorescent microscopy analysis of the brain section after the delivery of fluorescent-labeled PMO. Fluorescent-labeled PMO and BLs were intravenously injected into mice, and then the right hemispheres were exposed to 3.5 MHz pulsed HIFU conditions (intensity: 1.5 kW/cm^2^, exposure time: 30 s). Each brain was dissected, and the brain sections were sliced at 6 µm (*n* = 3). The nuclei were stained with DAPI. Each specimen was observed using fluorescent microscopy. Magnification: 200×. BL: Bubble liposomes.

It is known that Duchenne muscular dystrophy (DMD) is caused by the absence of dystrophin, which has crucial functions in both muscle and brain. Recently, 2′-*O*-methyl phosphorothioate RNA and phosphorodiamidate morpholino oligomers (PMOs) have been widely utilized in exon-skipping therapies for DMD, which has a nonsense mutation in the dystrophin gene. Once the oligonucleotides hybridize to the exon, including the nonsense mutation in the dystrophin gene, the exon that includes the nonsense mutation is skipped, which leads to the recovery of the deficient-dystrophin gene expression. Thus, the oligonucleotides have demonstrated usability in two independent clinical trials [32,33,34]. In particular, skipping efficiency of exon 23 from transcripts of dystrophin gene by treatment with PMOs is significantly higher than that of treatment with 2′-*O*-methyl phosphorothioate antisense oligonucleotide. To improve the delivery efficiency of PMOs into DMD muscle, we have demonstrated a novel method for delivering a PMO intramuscularly into the skeletal muscle in a DMD model mouse by using a combination of BLs and ultrasound exposure, which leads to significantly recovered dystrophin expression [35]. It has been reported that the recovery of the dystrophin expression in the brain of a DMD model mouse is critical for the amelioration of the altered neural functions in DMD [36,37]. However, it has been noted that naked AONs (2′-*O*-methyl phosphorothioate antisense oligonucleotide or PMO) do not cross the BBB or recover the lack of dystrophin in the brain following systemic injection of their AONs [38]. Therefore, it is important to develop a method that permits delivery of AONs (e.g., 2′-*O*-methyl phosphorothioate antisense oligonucleotide or PMO) across the BBB. For these reasons, we tried to deliver PMO by using a combination of BLs and HIFU. To determine the accumulation of PMO in the whole brain, fluorescent-labeled PMO and BLs were intravenously injected into mice, and the right hemispheres were exposed to HIFU conditions (intensity: 1.5 kW/cm^2^, exposure time: 30 s). Each tissue was corrected three hours after the injection, and the fluorescence intensity was observed by a Maestro *in vivo* imaging system. Subsequently, the brain tissue was sectioned using a brain slicer (divided with 2 mm slices), and the tissues were examined using an *in vivo* fluorescence imaging system. As shown in Figure 6A, the localized fluorescence intensity was detected in only the right hemisphere at the focused site, but no fluorescence intensity was detected in the left hemisphere at the non-focused site. Furthermore, the accumulation of fluorescently labeled PMO in the right hemisphere at the focused site was also confirmed by fluorescent microscopy as shown in Figure 6B. This result suggests that PMO can be systemically delivered into the limited brain tissue by the combination of BLs and HIFU. If PMO-loaded Bubble liposomes can be developed, a more efficient delivery of PMO into the brain may be achieved by using the combination of the BLs and HIFU.

### 3.3. Delivery of Plasmid DNA into the Brain Using a Combination of BLs and HIFU Exposure

**Figure 7:**
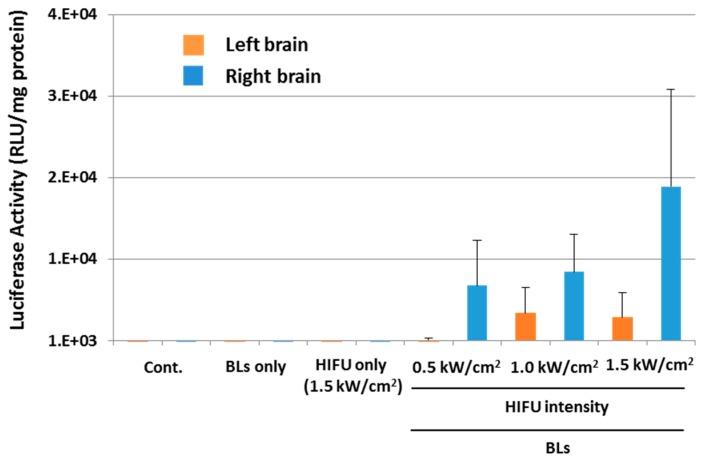
Delivery of plasmid DNA into the brain using the combination of BLs and HIFU exposure. Plasmid DNA encoding the luciferase gene and BLs were intravenously injected into mice, and the right hemispheres were exposed to 3.5 MHz pulsed HIFU conditions (intensity: 0.5–1.5 kW/cm^2^, exposure time: 30 s). Each tissue was corrected 24 h after the treatment, and the luciferase gene expression was determined. The activity is indicated by the relative light units (RLU) per mg of protein. Each value represents the mean S.D. (*n* = 3). BL: Bubble liposomes.

**Figure 8:**
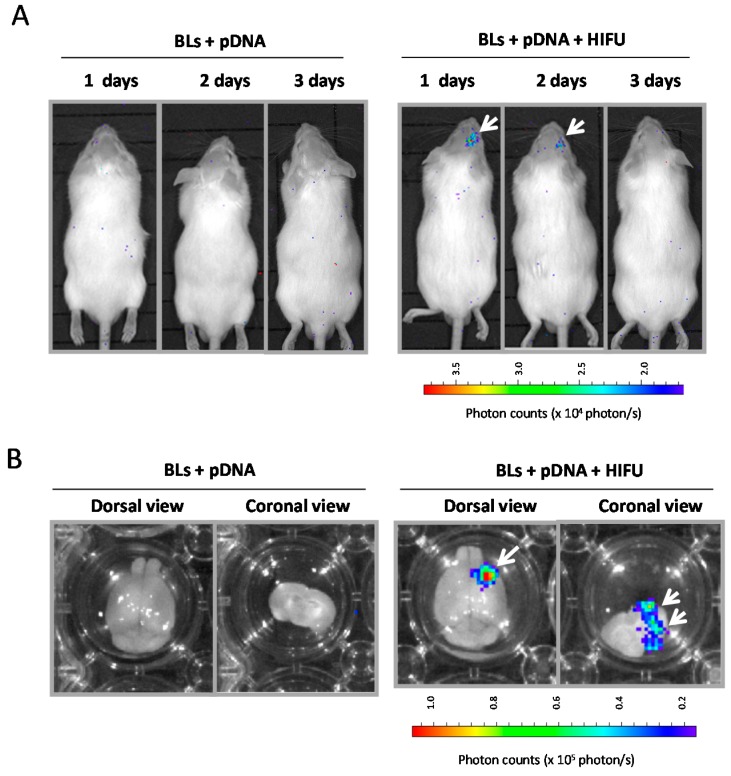
*In vivo* luciferase imaging after the delivery of plasmid DNA into brain using a combination of BLs and HIFU exposure. (**A**) *In vivo* luciferase imaging over three days. (**B**) *Ex vivo* luciferase imaging. Plasmid DNA encoding the luciferase gene and BLs were intravenously injected into mice, and then the right hemispheres were exposed to 3.5 MHz pulsed HIFU conditions (intensity: 1.5 kW/cm^2^, exposure time: 30 s). The luciferase expression after transfection into the brain treated with pDNA plus BLs, or pDNA plus BLs plus HIFU exposure, was observed with *in vivo* or *ex vivo* luciferase imaging by an IVIS system (*n* = 3). The white arrows indicate the region of luciferase expression.

We next examined whether the combination method of BLs and HIFU is a useful tool for the delivery of plasmid DNA. Plasmid DNA encoding the luciferase gene and BLs were intravenously injected into mice, and the right hemispheres were exposed to HIFU conditions (intensity: 0.5–1.5 kW/cm^2^, exposure time: 30 s). Each tissue was corrected 24 h after the treatment, and the luciferase gene expression was determined (Figure 7). The results showed that relatively higher gene expression could be observed by using the combination of BLs and HIFU, with an intensity of 1.0 or 1.5 kW/cm^2^, compared with the other treatment groups. We also observed the luciferase gene expression area in the whole body using an *in vivo* luciferase imaging system for three days after the delivery of plasmid DNA into the brain using a combination of BLs and HIFU exposure (Figure 8). Gene expression was detected for only two days after the delivery using the BLs and HIFU exposure (Figure 8A). As a result, the gene expression was restricted to the area of HIFU exposure. Gene expression was restricted to the area of HIFU exposure by *in vivo* and *ex vivo* luciferase imaging (Figure 8A,B). In contrast, no signal in the whole body or *ex vivo* imaging was observed in the group with pDNA plus BLs without HIFU exposure. This finding suggests that the combination of BLs and HIFU exposure facilitated efficient transfection of pDNA into the brain tissue due to the induction of cavitation. Although there is room for improving the gene expression level, the combination method of BLs and HIFU may become a useful tool for the delivery of plasmid DNA. Recently, it has been reported that cationic lipid-containing microbubbles loaded with pDNA enhanced gene transfection *in vitro* and *in vivo* [39,40,41]. However, it is thought that these microbubbles are insufficient to reach deeper tissues because their microbubbles have a size of 1–5 μm. In this view, we have developed cationic lipid-containing BLs that are capable of loading negatively charged molecules, such as plasmid DNA, siRNA, or miRNA [29,42,43,44], which led to their efficient delivery via systemic injection and therapy in a hind-limb ischemia model. Therefore, pDNA-loaded BLs combined with HIFU can be used to deliver pDNA into the brain, which is a more efficient delivery and enables a therapeutic system of gene therapy in CNS diseases.

## 4. Conclusions

In this study, we showed that the method of combining BLs and HIFU together serves to increase the permeability of the BBB and thereby enable antisense oligonucleotides or genes to be delivered to the focused brain site. To further enhance the delivery efficacy of nucleic acids (antisense oligonucleotides or gene expressing plasmid DNAs) for BBB component cells or brain parenchymal cells, it is important to accumulate nucleic acids-loading BLs at the BBB component cells. The subsequent intracranial HIFU exposure allows the nucleic acids to be delivered into the BBB component cells or cross the BBB into brain parenchymal cells. It would be easy to prepare a liposome that is tethered to targeting molecules (e.g., peptide, antibody, aptamers). We have successfully developed targeted BLs modified with peptides [45]. Therefore, in future experiments, if surface-modified BLs with specific targeting molecules for the brain, e.g., LDLR-related protein, rabies virus glycoprotein, RVG29, or ApoE can be developed [46,47,48,49,50], the safe and efficient delivery of nucleic acids (antisense oligonucleotides or gene expressing plasmid DNAs) may be achieved by using the brain-targeting BLs combined with HIFU exposure.
